# Gender differences and gender convergence in alcohol use over the past three decades (1984–2008), The HUNT Study, Norway

**DOI:** 10.1186/s12889-016-3384-3

**Published:** 2016-08-05

**Authors:** Grete Helen Bratberg, Sharon C Wilsnack, Richard Wilsnack, Siri Håvås Haugland, Steinar Krokstad, Erik Reidar Sund, Johan Haakon Bjørngaard

**Affiliations:** 1 HUNT Research Centre, Department of Public Health and General Practice, Faculty of Medicine, Norwegian University of Science and Technology (NTNU), Forskningsveien 2, 7600 Levanger, Norway; 2 Department of Research and Development, Levanger Hospital, Nord-Trøndelag Hospital Trust, Levanger, Norway; 3 Department of Psychiatry and Behavioral Science, University of North Dakota, School of Medicine & Health Sciences, Grand Forks, USA; 4 Department of Psychosocial Health, Faculty of Health and Sport Sciences, University of Agder (UiA), Kristiansand, Norway

**Keywords:** Alcohol, Drinking patterns, Gender differences, Change in gender differences, Gender convergence

## Abstract

**Background:**

To examine changes in men‘s and women’s drinking in Norway over a 20-year period, in order to learn whether such changes have led to gender convergence in alcohol drinking.

**Methods:**

Repeated cross-sectional studies (in 1984–86, 1995–97, and 2006–08) of a large general population living in a geographically defined area (county) in Norway. Information about alcohol drinking is based on self-report questionnaires. Not all measures were assessed in all three surveys.

**Results:**

Adult alcohol drinking patterns have changed markedly over a 20-year period. Abstaining has become rarer while consumption and rates of recent drinking and problematic drinking have increased. Most changes were in the same direction for men and women, but women have moved towards men’s drinking patterns in abstaining, recent drinking, problematic drinking and consumption. Intoxication (among recent drinkers) has decreased in both genders, but more in men than in women. The declines in gender differences, however, were age-specific and varied depending on which drinking behavior and which beverage was taken into account.

**Conclusions:**

There has been a gender convergence in most drinking behaviours, including lifetime history of problem drinking, over the past 2–3 decades in this Norwegian general population, but the reasons for this convergence appear to be complex.

**Electronic supplementary material:**

The online version of this article (doi:10.1186/s12889-016-3384-3) contains supplementary material, which is available to authorized users.

## Background

Europeans have been called “world champions” at drinking and smoking [1]. Although the consumption in Norway has been fairly low compared to most other European countries, the country has previously been given a high harm score associated with alcohol drinking [2] and drinking patterns have changed with increasing levels of consumption [3, 4]. The observed increase in women’s drinking has given cause for concern not only in Norway [5], but in many countries e.g., Keyes et al. [6]. Women may drink alcohol with less restraint than ever, but will simultaneously be exposed to higher levels of alcohol-related harm and alcohol problems [7]. It remains largely unclear however, to what extent gender differences in drinking are changing [8].

Traditionally, men have consumed more alcohol over more frequent drinking occasions and exceeded women in rates of heavy drinking and adverse drinking consequences, while women consistently have been more likely to be life-time abstainers [9, 10]. The size of the gender gap in drinking varies across geographical settings, but is suggested to be more influenced by men’s drinking behaviors than women’s [11]. Linkages between male and female drinking behaviours, however, are uncertain and complicated. Most likely the differences are due in part to both biological differences and culturally defined gender-specific roles, but other societal patterns may also be influential [8]. One such societal pattern may be the degree of gender inequality: it has been suggested that gender differences in drinking are smaller in countries with greater gender equality [12, 13]. The smallest gender differences in drinking have been found in the Nordic countries, including Norway, and the largest in countries with developing economies [14]. Sociodemographic factors such as age, education, employment, marital status and parenthood are all suggested to influence the way men and women drink, but may act differently on gender differences in different societies [15].

Although gender roles and equality may have changed in modern societies, gender differences in drinking behaviour continue to be substantial in most cultures [10]. However, there have been some signs of gender convergence in alcohol drinking and in problematic use [16–24]. By convergence we mean that differences between men‘s and women’s drinking behavior (in prevalence, frequency, or quantity) have grown narrower over time. Such narrowing of gender differences could result from a variety of increases and/or decreases in men‘s and women’s drinking behavior. A population study from New Zealand showed gender convergence related to a range of measures during the period from 1995 to 2000, but increases in women’s drinking were most apparent among women 20–39 years old [22]. In the US, gender convergence in level of alcohol consumption, binge drinking, and alcohol dependence has been more evident in younger age groups than in older [18].

The gender convergence observed in Nordic countries during recent decades may partly be due to the increasing wine consumption [25, 26] in these traditionally beer- or liquor-drinking societies. It has been suggested that gender differences are smaller when new beverages are introduced, and many women seem to prefer wine to other alcoholic beverages [27, 28].

Gender convergence has not been found for all age groups or all drinking patterns in all populations studied [29, 30]. It is important to understand how any such convergence in different societies is related to general changes in consumption levels, and to what extent such convergence can be explained by increases or decreases in women’s drinking versus men’s drinking. Data on these historical changes are scarce. One such data set is provided by the Norwegian “Nord-Trøndelag Health Study” (HUNT), conducted as three cross-sectional surveys between 1984 and 2008, within a demographically and geographically stable adult population in Northern Europe. The present study aims to examine changes in gender differences in drinking patterns between HUNT1 (1984–86), HUNT2 (1995–97), and HUNT3 (2006–08).

## Methods

### Sample

Every citizen aged 20 years or older in Nord-Trøndelag County in Norway was invited to participate in the HUNT study, which consisted of both questionnaires and clinical examinations. Three HUNT surveys were completed in three consecutive decades and for each new survey all eligible previous respondents were invited to participate. HUNT1 (1984–1986) had 77 216 participants (86 404 invited, 89 % response rate), HUNT2 (1995–1997) had 65 215 participants (93 898 invited, 71 % response), and HUNT3 (2006–2008) had 50 624 participants (93 860 invited, 54 % response) (Table 1). Most of the decline in participation rates is due to increasing attrition among younger and older citizens, and in younger men in particular [31–33]. Participation rates in middle aged men and women (39–79 years) were stable. Of the total respondents (*N* = 110 060), 43 % participated in two surveys and 25 % in all three surveys [32]. In many respects (e.g., geography, economy, industry, sources of income, age distribution, morbidity and mortality), Nord-Trøndelag County is considered fairly representative of Norway [31]. More comprehensive information about the HUNT study is available elsewhere [31–33]. The abbreviations H1 (HUNT1), H2 (HUNT2) and H3 (HUNT3) are used in the Methods and Results sections.

**Table 1:** Characteristics of men (M) and women (W) in the Norwegian HUNT Study, the HUNT1 (1984–86), HUNT2 (1995–97) and HUNT3 (2006–2008) surveys

	HUNT1			HUNT2			HUNT3		
	W	M	Total N	W	M	Total N	W	M	Total N
Questionnaire 1 (Q1) n=	39390	37826	77216	34653	30562	65215	27676	22948	50624
Questionnaire 2 (Q2) n=	32776	31174	63950	30315	25137	55452	23154	18140	41294
Agegroups	%	%	n	%	%	n	%	%	n
20-29	8	8	12557	7	6	8826	5	4	4467
30-39	11	11	16261	9	8	11549	8	6	6842
40-49	8	8	12099	11	10	13568	11	9	9989
50-59	7	7	11404	9	8	11184	12	11	11404
60-69	8	8	12535	7	7	9064	10	9	9778
70-79	6	5	8819	7	6	8103	6	5	5739
≥80	3	2	3541	3	2	2921	3	2	2405
*Total*	*51*	*49*		*53*	*47*		*55*	*45*	
Mean age (years)	50,2	48,9	49,5	50,3	49,8	50,0	52,8	53,6	53,1
Education %									
Primary	46	39	43	33	28	31	23	20	21
Secondary	40	48	44	45	54	49	48	58	53
College/university	14	13	13	22	18	20	29	22	26
Marital status %									
Partnered	82	73	78	72	65	69	68	65	67
Single	18	27	22	28	35	31	32	35	33

### Procedures

In all surveys, the data collection followed a standard procedure. Letters of invitation were mailed some weeks before the time of appointment and included a questionnaire (Q1) and a brochure with the aims of the study and information about the examinations and procedures. At the screening station, this initial questionnaire was collected from the participants before they underwent different physical examinations. In all three surveys, the participants were given a second questionnaire (Q2) which they were instructed to complete at home and return by mail in pre-addressed stamped envelopes. In H1, all alcohol measures were located in Q2. In H2 and H3, Q1 included frequency and volume measures and Q2 included the CAGE index. The response was higher for Q1 than for Q2 (Table 1).

### Measures

Questions about alcohol drinking were designed to obtain information about drinking patterns as well as volume consumed during a specific time period; the questions were previously used in similar screening surveys in Norway [34]. However, the type of questions, question wording and reference periods all varied to some extent across the H1, H2 and H3 surveys. All surveys included drinking-frequency questions; H2 and H3 also included measures of quantities consumed, while the H1 and H3 surveys included questions about intoxication. More details about questions and definitions of measures are presented in a Additional file 1 supplementary overview table. The web-link to the HUNT questionnaires http://www.ntnu.edu/hunt/data/que.

Estimates of abstaining, recent drinking and lifetime problematic drinking were based on total survey samples, while estimates of intoxication and alcohol consumption were based on those classified as recent drinkers. ***Recent drinking*** was defined as at least one drinking occasion during the last 14 days in H1. In H2 and H3 those who had consumed alcohol during the past four weeks and simultaneously reported how much they usually consumed during a period of 2 weeks were classified as recent drinkers. In H1 and H2, those who reported total abstention were classified as ***abstainers***. Since H3 included the response category “never in life consumed alcohol” (4,1 %), we merged these abstainers with those who never had consumed alcohol during past 12 months (4,8 %) to make more comparable groups. ***Intoxication*** was measured in H1 and H3, but not in H2. Estimates were based on recent drinkers in both survey samples.

In H2 and H3 the prevalence of ***lifetime problematic drinking*** was assessed by the use of the CAGE index, which is one of the most widely validated screening tools for detecting alcohol abuse and dependence in primary care [35, 36]. The standard CAGE consists of four items: 1) Have you ever felt that you ought to Cut down on your drinking?; 2) Have people Annoyed you by criticizing your drinking?; 3) Have you ever felt bad or Guilty about your drinking?; and 4) Have you ever had a drink first thing in the morning to steady your nerves or to get rid of a hangover (Eye-opener)? In the HUNT questionnaires the phrasing of item 2 differed slightly from the English version: “Has someone ever criticized your drinking?” The CAGE has demonstrated high test-retest reliability (0.80-0.95) [37]. The CAGE scores had good concurrent validity and adequate psychometric properties in the H2 survey (Skogen et al., 2011), with a linear relationship of H1 and H2 excessive consumption with H2 CAGE-scores. For the present analyses we used the CAGE as a dichotomous measure with the standard screening cut-off of two or more [37].

Calculation of *annual volume of alcohol consumption* was based on beverage-specific usual quantities (number of units). Participants were asked (in H2 and H3) to separately report the amount of beer, wine, or liquor they usually consumed during a period of two weeks, indicated by bottles of beer and glasses of wine or liquors. Alcohol consumption was then calculated in liters of pure alcohol per year. A standard bottle of beer (33 cl) was assumed to contain 4,5 % ethanol, a glass of wine (12 cl) 12 % ethanol and a shot of liquor (2 cl) 40 % ethanol. Estimates of beverage-specific and total alcohol consumption were based on recent drinkers who reported any intake of alcohol during the past two weeks.

Age, education level and marital status may account for some apparent gender differences and changes in gender differences in alcohol consumption [15], so these variables were included in all multivariate models. Information about education level was obtained from the Norwegian education register (Statistics Norway), and marital status was obtained from the National Population Registry (SSB). Education had 8 levels, with university graduation as the highest. Since it not was possible to make distinctions between married and cohabiting partners, marital status was dichotomized into “partnered” (married or cohabitating) or “single”.

### Statistics

Prevalence rates, gender differences and changes in gender differences in abstaining, recent drinking, intoxication and problematic drinking were calculated for the total sample and for 10-year age groups. Since a number of the individuals in this study participated in two (H1/H2 = 47 316; H2/H3 = 37 071) or three of the surveys (27 992), these observations are considered clustered or non-independent. To account for this dependency we fitted marginal and mixed models for the binary and continuous outcomes respectively. For the former we utilized generalized estimating equations (GEE). More specifically, we specified models, with a logit link function, the correlation structure was set to unstructured, and we selected robust standard errors. Gender differences in alcohol drinking are reported as odds ratios (OR) with 95% confidence intervals (95 % CI). For the continuous outcomes (total and beverage-specific alcohol consumption) we specified linear mixed models and report unstandardized coefficients with 95% confidence intervals. In order to test for changing gender differences between surveys we included an interaction term between gender and survey. All models were adjusted for continuous age, age squared, education level and marital status. P-values ≤0.05 (two-tailed) were considered to be significant. The multivariable analyses were conducted in STATA version 13 (StataCorp LP, College Station, Texas, USA).

## Results

Table 1 shows some basic characteristics of the respondents in the three surveys. The proportion with university or college graduation increased from 13 % in 1984–1986 (H1) to 20 % in 1995–97 (H2) and 26 % in 2006–2008 (H3), and was more common among women (29 %) than among men (22 %) in the third survey. The proportion of adult women and men living with partners (married or cohabiting), decreased from 78 % in 1984–1986 to 67 % in 2006–2008, and was similar for men (65 %) and women (68 %) in H3. According to Q1 responses (Table 1) the proportion of women of the total sample slightly increased from 51 % (H1) to 53 % (H2) to 55 % in the H3 survey. Respondents were on average slightly older in H3 (53,1 years), than in H2 (50,0 years) and H1 (49,5 years).

### **Abstaining**

Rates of abstaining have declined in both genders (Table 2), with greater overall decline in women than in men (gender difference change; p-value < .001), but not in all age groups (Table 3). Between H2 and H3 there was gender divergence in two age groups: abstinence rates increased in younger women (20–39 years) but not in men, and in the oldest age group (80+) men’s abstinence decreased more than women’s. In age groups 40–79, however, there was gender convergence because women’s abstinence declined more than men’s.

**Table 2:** Prevalence rates, gender differences^a^ (OR, 95 % CI) and change in gender differences^b^ over time in abstaining, recent drinking, lifetime problematic drinking and intoxication (recent drinkers) as reported in the HUNT1-2-3 surveys (1984–2008)

	Total survey samples			Recent drinkers
	Abstaining^c^	Recent drinking^d^	Problematic drinking^e^	Intoxication^f^
H1 1984-1986	*N* = *61528*	*N* = *61528*	*No data*	*N* = *25123*
All	12	42		40
Women	17	30		24
Men	7	55		49
*Absolute differences* %	*10*	*25*		*25*
Multivariate adjusted gender differences OR (95%CI)	0.38 (0.36-0.40)	3.03 (2.68-2.86)		3.93 (3.69-4.19)
H2 1995-97	*N* = *63349*	*N* = *63349*	*N* = *46493*	*No data*
All	13	56	7	
Women	17	47	3	
Men	8	66	13	
*Absolute differences*%	*9*	*19*	*10*	
Multivariate adjusted gender differences OR (95%CI)	0.43 (0.41-0.45)	2.42 (2.34-2.51)	5.31 (4.89-5.78)	
Gender differences change^b^:	*P* < 0.001	*P* < 0.001		
H3 2006-08	*N* = *48969*	*N* = *48802*	*N* = *37174*	*N* = *36559*
All	9	74	9	20
Women	11	67	4	13
Men	6	81	14	26
*Absolute differences*%	*5*	*14*	*10*	*13*
Multivariate adjusted gender differences OR (95%CI)	0.49 (0.46-0.53)	2.25 (2.15-2.35)	4.22 (3.89-4.57)	2.88 (2.72-3.05)
Gender difference change^b^:	*P* < 0.001	*P* < 0.001	*P* < .0.001	*P* < 0.001

^a^Gender differences (survey- specific) reported as odds ratios, with 95% confidence intervals (ORs, 95 % CIs, adjusted for age, level of education, marital status with women as references); ^b^Gender differences change: *p*-value for interaction term between gender and survey (H1 and H2 = reference). ^c^Abstaining: H1 and H2 = those who reported total abstinence from alcohol; H3 = those who never in lifetime or during last year had consumed alcohol ^d^Recent drinking: H1 = At least one drinking occasion - past 14 days; In H2 and H3 those who reported at least one drinking occasion - past 4 weeks and also any usual amount of alcohol consumption during 2 weeks were classified as recent drinkers. ^e^Lifetime problematic drinking in H2 and H3: claims at least 2 out 4 confirming answers using the CAGE index. ^f^Intoxication among recent drinkers: H1 = past 14 days; H2 not measured; H3 = past 4 weeks

**Table 3:** Gender differences^a^ and change in gender differences in **alcohol abstaining** ^b^ in 10 year-age groups in proportions (%), gender ratios (men versus women) and ratio differences (HUNT1 versus HUNT2 versus HUNT3)

	1984-86 HUNT1 *N* = *61528*			1995-97 HUNT2 *N* = *63349*			2006-08 HUNT3 *N* = *48969*			1984-2008 Change in gender difference^c^	
Age	W %	M %	*M*/*W* *ratio*	W %	M %	*M*/*W* *ratio*	W %	M %	*M*/*W* *ratio*	HUNT 1 versus 2	HUNT 2 versus 3
20-29	4	3	0,77	6	5	0,78	8	4	0,53	−0,01	0,25
30-39	7	4	0,55	6	4	0,73	8	4	0,44	−0,18	0,29
40-49	10	5	0,45	8	4	0,56	5	3	0,64	−0,11	−0,07
50-59	17	7	0,40	14	8	0,54	7	5	0,62	−0,14	−0,08
60-69	27	10	0,37	25	11	0,43	13	7	0,57	−0,06	−0,14
70-79	37	15	0,40	42	18	0,44	26	12	0,47	−0,03	−0,04
80+	45	21	0,47	49	27	0,55	39	21	0,53	−0,09	0,03
*Total*	17	7	0,42	17	8	0,50	11	6	0,55	−0,08	−0,05

^a^All gender differences by survey and age-group were statistically significant (*P* < .05)

^b^Abstaining; H1 and H2 = those who reported total abstinence from alcohol; H3 = those who never in lifetime or ever during last year had consumed alcohol

^c^Gender ratio differences: positive change indicates divergence, and negative indicates convergence

### **Recent drinking**

Along with the general decline in abstaining, the proportion of recent drinkers increased from 42 % (H1) to 56 % (H2) to 74 % (H3) and in both genders (Table 2). The absolute and relative gender differences decreased and the multivarible adjusted results (Table 2) suggest a greater increase in women than in men (gender difference change; p-value < .001). Table 4 shows that gender differences in recent drinking decreased in all age groups and that gender ratios in the most recent survey (H3) were close to 1 in many age groups.

**Table 4:** Gender differences^a^ and change in gender differences in **recent drinking** ^b^ in 10 year-age groups, in proportions (%), gender ratios (men versus women) and ratio differences (HUNT1 versus HUNT2 versus HUNT3)

	1984-86 HUNT1 *N* = 61528			1995-97 HUNT2 *N* = 63349			2006-08 HUNT3 *N* = 48802			1984-2008 Change in gender difference^c^	
Age groups	W %	M %	M/W ratio	W %	M %	M/W ratio	W %	M %	M/W ratio	HUNT 1 versus 2	HUNT 2 versus 3
20-29	49	74	1,51	66	80	1,21	73	86	1,18	0,30	0,03
30-39	46	68	1,51	61	79	1,29	72	86	1,19	0,22	0,10
40-49	39	64	1,66	59	75	1,28	79	88	1,11	0,38	0,17
50-59	26	52	1,98	48	68	1,42	75	87	1,16	0,55	0,27
60-69	14	39	2,81	31	53	1,74	63	78	1,24	1.08	0,49
70-79	7	26	3,71	16	37	2,34	43	67	1,58	1.35	0,76
80+	3	17	5,14	8	23	2,84	27	47	1,74	2,30	1,07
Total	30	55	1,80	47	66	1,40	67	81	1,20	0,40	0,20

^a^All survey specific age-group gender differences were statistically significant (*P* < .05)

^b^Recent drinking: H1 = At least one drinking occasion - past 14 days; In H2 and H3 those who reported at least one drinking occasion - past 4 weeks and simultaneously their usual amount of alcohol consumption during 2 weeks, were classified as recent drinkers

^c^Gender ratio differences: positive change indicates convergence and negative indicates divergence

### **Alcohol consumption** (**volume**)

The mean annual alcohol consumption (among recent drinkers) increased slightly between H2 and H3, but not for all beverages. The average intake of wine nearly doubled (0.55 to 0.98 l), while the consumption of liquors declined (0.78 to 0.40 l), from 36 % to 17 % of all intake, from H2 to H3. Table 5 shows that mean consumption increased in both genders, but slightly more in women than in men (gender difference change; *P* = .029). The adjusted beverage-specific volume estimates (Table 5) suggest that while **beer** consumption increased among men, it slightly decreased among women during the same period (gender difference change: *P* < .001). The intake of **wine** increased in both genders, but more in women than in men (gender difference change: *P* < .001). The intake of **liquor** declined in both genders, but more in men than in women (gender difference change: *P* < .001). Table 6 shows that mean annual volume of alcohol consumption among recent drinkers increased in all age groups, except for women aged 30–39 years. There was a tendency of younger (20–29 years) and older women (60+) to have increased their annual consumption disproportionally to men (gender convergence). Conversely, men aged 30–49 years increased their intake slightly more than same-aged women (gender divergence).

**Table 5:** Gender differences in total^a^ and beverage-specific alcohol consumption (in liters of pure alcohol a year) in HUNT2 and HUNT3, in multivariate adjusted means and mean differences (with 95 % confidence intervals)^b^

	Liters of pure alcohol a year			
*N* = 35587	Total volume	Beer	Wine	Liquor
HUNT2 All	*2.18*	0.84	0.55	0.78
Women	1.59	0.50	0.70	0.39
Men	2.66	1.11	0.43	1.11
Mean gender differences (95 % CI)^b^	1.13 (1.09, 1.18)	0.67 (0.65,0.70)	−0.25 (−0.27, −0.22)	0.70 (0.68,0.72)
*N* = 36322				
HUNT3 All	*2.32*	0.85	0.98	0.40
Women	1.80	0.47	1.16	0.17
Men	2.83	1.41	0.80	0.63
Mean gender differences (95 % CI)^b^	1.08 (1.03, 1.12)	0.98 (0.95, 1.00)	−0.35 (−0.37,-0.33)	0.45 (0.43, 0.47)
Gender differences change^c^:	*P* = 0.029	*P* < .001	*P* < .001	*P* < .001

^a^Total consumption was the summarized beverage-specific intake reported by recent drinkers

^b^Means and mean differences were adjusted for age, education level and marital status

^c^Gender differences change: p-value for interaction term between gender and survey (H2 = reference)

**Table 6:** Gender differences^1^ and change in gender differences in **mean annual volume** of alcohol (in liters a year) by 10 year-age groups of recent drinkers^a^ in gender ratios (men’s means versus women’s means) and ratio differences (HUNT2 versus HUNT3)

	1995-97 HUNT 2 *N* = 35692		2006-08 HUNT 3 *N* = 36322		HUNT 2	HUNT 3	1995-2008 Change in gender differnces^b^
Age groups	W means	M means	W means	M means	M/W ratio	M/W ratio	HUNT 2 versus 3
20-29	1,75	3,54	2,18	3,89	2,02	1,78	0,24
30-39	1,47	2,67	1,47	2,78	1,82	1,89	−0,07
40-49	1,65	2,56	1,83	2,87	1,55	1,57	−0,02
50-59	1,67	2,49	1,96	2,93	1,49	1,49	0,00
60-69	1,41	2,29	1,78	2,63	1,62	1,48	0,15
70-79	1,34	2,07	1,50	2,25	1,54	1,50	0,04
80+	1,23	1,95	1,31	1,97	1,59	1,50	0,08
Total	1,59	2,66	1,80	2,83	1,67	1,57	0,10

^1^All gender differences by survey and age-group were statistically significant (*P* < .05)

^a^Estimates were based on recent drinkers who also reported any amounts of alcohol consumption

^b^Gender ratio differences: positive change indicates convergence and negative change indicates divergence

### **Intoxication**

Drinking to intoxication among recent drinkers was halved in H3 (20 %) compared to H1 (40 %) and became less prevalent among both men and women than it was at H1, with exception of the youngest women. The absolute gender differences decreased from 25 % to 13 % (Table 2) and the overall and multivariable adjusted results suggest an overall steeper decline in men than in women (gender difference change: *P* < .001, Table 2), but not in all age groups. Table 7 shows that women drinkers aged 20–29 years were more likely to report intoxication in H3 (55 %) than in H1 (42 %), while intoxication in same-aged men declined (gender convergence). In age groups 40–59 years, declines were steeper in women than in men (gender divergence) while in age groups 60+ declines were greater in men than women (gender convergence) (Table 7).

**Table 7:** Gender differences ^a^ and change in gender differences in drinking to **intoxication** (recent drinkers) ^b^ in 10 year-age groups in proportions (%), gender ratios (men versus women) and ratio differences (HUNT1 versus HUNT3)

	1984-86 HUNT1 *N* = 25123		2006-08 HUNT3 *N* = 36559		HUNT1	HUNT3	1984-2008 Change in gender differences ^c^
Age groups	W %	M %	W %	M %	M/W ratio	M/W ratio	HUNT 1 versus 3
20-29	42	73	55	69	1,76	1,24	0,52
30-39	25	55	20	43	2,21	2,20	0,02
40-49	18	45	12	32	2,54	2,71	−0,16
50-59	13	38	6	21	2,93	3,40	−0,47
60-69	6	25	3	12	4,60	4,21	0,51
70-79	4	15	1	7	3,50	4,76	−1,26
Total	24	49	14	27	2,02	1,97	0,04

^a^All gender differences in proportions by survey and age-group were statistically significant (*P* < .05)

^b^Intoxication among those classified as recent drinkers

^c^Gender ratio differences: positive change indicates convergence and negative change indicates divergence

### **Lifetime problematic drinking**

The prevalence of lifetime problematic drinking (CAGE scores of 2 or more) in the total sample increased from 7 % to 9 % between H2 and H3 (Table 2). The 10% absolute gender difference was similar for H2 and H3, but multivariable adjusted odds ratios suggest a higher increase in problematic drinking among women than men (gender difference change, p-value < .001). Table 8 shows that problematic drinking increased in all age groups of men and women and that the increase in problematic drinking was more pronounced in women than in men.

**Table 8:** Gender differences^a^ and change in gender differences in **lifetime problematic drinking** ^b^ by 10 year-age groups, in proportions (%), gender ratios (men versus women) and ratio differences (HUNT2 versus HUNT3)

	1995-97 HUNT2 *N* = 43 261		2006-08 HUNT3 *N* = 31 644		HUNT2	HUNT3	1995-2008 Change in gender differences^c^
Age groups	W %	M %	W %	M %	M/W ratio	M/W ratio	HUNT 2 versus 3
20-29	5	18	8	20	3,85	2,35	1,50
30-39	3	15	4	17	5,00	4,35	0,65
40-49	4	14	5	17	4,03	3,33	0,70
50-59	2	12	4	15	7,19	3,62	3,56
60-69	1	8	3	12	7,98	4,64	3,34
Total	3	13	4	14	4,99	3,58	1,41

^a^All gender differences by survey and age-group were statistically significant (*P* < .05)

^b^Problematic drinking; i.e. responded “yes” on two or more of four items of the CAGE index

^c^Gender ratio differences: positive change indicates convergence

## Discussion

This study suggests that alcohol drinking patterns among adults in Norway have changed in several ways over the past 2–3 decades. Abstaining has become rarer while consumption, recent drinking and lifetime problematic drinking have increased. Most changes were in the same direction for men and women, but women have moved towards men’s drinking patterns in abstaining, recent drinking, problematic drinking and in mean volume of consumption (liters a year). Among recent drinkers, intoxication has decreased in both genders, but more in men than in women. The declines we noted in gender differences, however, did not occur for all age x gender groups nor for all alcoholic beverages: they were age-specific and varied depending on which drinking behavior and which beverage was observed. For example, in the youngest age group, intoxication became less frequent in men and more frequent in women, indicating gender convergence. Some gender convergence in alcohol consumption was due to the greater decline in men’s than in women’s mean consumption of liquor.

### Changing drinking patterns

The overall changes in drinking patterns in this study are very much in accordance with previous Norwegian studies [3, 4, 38]. A range of secular changes in society may help explain the changing drinking patterns, and some of the most important conditions may be the improved family economy and the ongoing globalisation [26, 39]. Norway has historically kept a strict alcohol policy with high prices and restricted availability [40]. Although prices have not decreased in real terms, the increase in general spending power has made alcohol “less expensive” for most Norwegians. Simultaneously the access to “cheaper” alcoholic beverages through cross-border and international tax-free shopping has expanded dramatically [41]. The number of alcohol outlets have increased and the time that pubs and other places that serve alcoholic beverages are open have expanded rather than declined [42].

Extended international travelling has not only made alcohol more available, but has apparently also influenced the drinking culture. In this study most of the consumption of liquors seems to have been “replaced” by wine drinking that increased from 25 % to almost half of all consumption (42 %) within the same period. Correspondingly, national sales statistics show that wine consumption increased 18-fold from 1960 to 2005 and made up 37% of all alcohol intake in 2012, reflecting the change in people’s beverage preferences [41]. Despite the fact that alcohol consumption has increased, all changes in this study should not be considered disadvantageous. Norway has previously been given a high harm score associated with alcohol drinking [2] and any changes towards more moderate use should be considered beneficial. The observed decline in drinking to intoxication may at least indicate a trend toward less heavy episodic drinking.

### Changing gender differences in alcohol drinking

Our results suggest that gender differences have decreased for many alcohol measures, but that the size of the decrease varied depending on age group. This is in accordance with most previous studies of gender differences, but Norwegian men and women may constitute an exception in some aspects of alcohol drinking. Results from the cross-cultural and multinational GENACIS study showed that Norwegian gender ratios differed from most of the other countries included, although some differences were restricted to the youngest age group (18–34 years) [10]. The GENACIS analyses found that Norwegian women were as likely as men to be current drinkers (in all age groups) and in the youngest age group there were no gender differences in high-frequency drinking.

In studies of changing drinking patterns, there is an ongoing debate whether decreasing gender differences are due to age, period or cohort effects. Although these factors are overlapping and time effects always can be interpreted as a combination of cohort and age effects, birth cohort effects may reflect historical influences and experiences that have imprinted the life-course attitudes and behaviors of individuals born at particular times. We have not investigated possible birth cohort effects in this study, but the idea that gender convergence is most evident in younger cohorts [6], is partly consistent with our finding a decline in gender differences and a narrowing of the gender gap in the youngest age group (20–29 years). On the other hand, our findings also suggest a more consistent decline in most gender differences in most age groups of adults and for most alcohol measures during the past 2–3 decades. A somewhat similar consistency among adults was reported in a study from New Zealand between 1995 and 2000, but in that study all changes in gender differences (in adults 20+) were explained by increases in women’s drinking [22].

Some research has hypothesized that gender differences in drinking may become smaller as women’s rights and social status improve relative to men’s. For example, Rahav et al., [43] found that in countries with lower levels of gender inequality and greater gender empowerment, gender differences in current drinking were smaller. However, these data were cross-sectional and so did not measure how changes in gender inequality might be related to changes in gender ratios of drinking. Gender equality is not synonymous with increased alcohol-related harm among women [44], but associations between women’s social status and alcohol drinking may be complicated and may be very different in higher-income versus lower-income countries [45]. They may also differ between women at high and low economic levels within a country [7]. Bergmark (2004) suggested on basis of Swedish data that social background factors may play a lesser role for gender differences in alcohol use today than during the 1970s [16]. This may be true for Sweden and other Nordic countries, given the combination of high levels of gender equality and social welfare, but not necessarily for other countries.

Only a few studies of gender convergence have focused on men’s drinking, but there is some evidence of changes towards more moderate drinking in men. For example, Neve et al. reported gender convergence that was explained by a decline in more highly educated men’s consumption [23]. Saelan et al. followed a Danish birth cohort and found decreasing consumption in men and the reverse in women [46]. In a study by Bloomfield et al., there was a tendency of men in both Germany and Switzerland (not in Finland) to have decreased more than women in rates of hazardous drinking between 1984 and 1992 [17]. These findings towards more moderate drinking in men, have to our knowledge not attracted much attention. This may reflect that, at least in part, more attention has focused on changes in women’s alcohol use than in men’s. The one-sided and negative focus on increases in women’s drinking has been criticized as a social construct that serves to blame women [47]. More important perhaps, a hypothesis based solely on changes in women’s drinking pays no attention to ways that gender convergence in drinking may occur because of changes in men’s drinking, or changes in both genders.

Although most changes in our study were in the same direction for men and women there were some desirable changes in men’s drinking that should be emphasized. The halving of intoxication in men (among recent drinkers) should be noted, since male intoxication has consistently been associated with substantial alcohol-related harm [9, 10]. Men’s changes in beverage preferences from liquors to beer and wine consumption may also be considered a positive change, in terms of shifting to beverages with lower alcohol concentration.

Another “favorable finding” was observed in women aged 30–39 years, which is the period most (Norwegian) women give birth to and bring up children. Between HUNT2 (1995–97) and HUNT3 (2006–08) the average consumption in this age group was stable, abstaining increased, while drinking to intoxication became more rare. Problematic drinking increased, but seemingly to a lesser extent than in other age groups. Future research should investigate to what extent the culture of childbearing and childrearing in Norway may have changed to discourage alcohol consumption among women at this stage of life.

Despite the possible trend towards more moderate use of alcohol (decline in intoxication) in this Norwegian population, rates of lifetime problematic drinking increased in both genders, slightly more in women than in men. While the incidence of developing alcohol problems or dependence during one’s lifetime in men seems to be stable in many countries, a recent longitudinal Swedish study suggested that the incidence among women increased between 1972 and 1997 [48]. Another recent Icelandic study reported gender convergence in discharge diagnosis of alcohol use disorders among psychiatric patients [24]. These changes may be of great concern and opposite to what the World Health Organization has appealed for: a reduction in the social, medical and economic costs of excessive alcohol drinking [49].

### Strengths and limitations

One advantage of the HUNT surveys is that the county surveyed, situated in the middle part of Norway has a very stable and homogenous population, with little migration and few cultural disparities related to religion and ethnicity. The repeated cross-sectional surveys of the general population located within the same geographic area, strengthen the likelihood of reliable estimates of change and comparisons across age groups and genders. The demographic stability of the county also lends strength to the idea that changes observed in drinking behaviour were cultural, and not the result of demographically different persons moving into and out of the county. The Nord-Trøndelag population has been shown to be very similar to the general population of Norway in many health aspects [31, 33] https://www.khs.fhi.no/en/health-in-the-municipalities/. Since this county lacks major cities, estimates of change in alcohol consumption may not correspond to the changes in the most urban parts of the country, but they are very much in accordance with changes reported in previous studies of adults [4, 38, 41], based on national representative samples between 1973–2004.

As in general population surveys elsewhere in the world [50] the participation rate in HUNT decreased considerably since the 1980s, especially in younger (20–39 years) and older age groups (80+) and more in men than in women. In HUNT3, women constituted 55 % of the study population. Participation rates among middle-aged men and women (50–79 years) have been stable and of less concern. In HUNT3 68 % of eligible men and 74 % of eligible women aged 60–69 years participated in the study [33].

Of more concern in studies of change, perhaps, is the increasing gender imbalance in attrition and whether e.g. male responders in HUNT have become less representative of the general population. According to a previous non-response study of HUNT1 and HUNT2, attrition was moderately associated with both abstaining and heavy drinking, but was not considered a major cause of nonresponse after taking other characteristics into account [51]. Although the gender imbalance in response rates may explain some of the observed decrease in gender differences, it is important to note that gender ratios of intoxication and mean annual consumption were based on recent drinkers and not total samples. Changing gender ratios of these two measures are therefore not a consequence of more women drinking or fewer women abstaining.

Due to the substantial decline in participation rate in HUNT3, a thorough nonparticipation study was conducted [33], including questionnaire data from 6922 HUNT non-responders, National Registries data and data from General Practitioners in the county. Non- responders were (among other factors) characterized by lower socioeconomic status, higher mortality and higher prevalence of chronic disorders. The study gives no direct answer to the question of why more women than men responded in HUNT3, but among those younger than 40 years of age, the main reasons for non-response in both genders were “had no time/ inconvenient” and “got no invitation”. Of more importance however, there were no differences in alcohol drinking (i.e., drinking 2–3 times a week or more often) or in daily smoking between responding and non-responding men and women aged 20–39 years. Lifestyle factors accounted only for a small fraction of the observed underestimation of a range of outcomes (e.g., morbidity and mortality) after taking social status (SES) into account. In this study, educational level was used as proxy of SES and possible confounding variable.

On the other hand, if gender imbalance in attrition has to do with gender specific changes in educational level, the survey samples of men and women may have become less representative for their source populations, and samples less comparable over time. According to available national statistics of men and women aged 30–39 years http://www.norgeshelsa.no), higher education (high school or university) was slightly more common among female responders in HUNT responders, than in this region (3 counties) and in the country, while the corresponding proportion in HUNT male responders was lower than in the country, but similar to that in the region. More important perhaps, these differences have not changed over time and thus not suggested to represent a selection bias. Although the increasing attrition, in younger men in particular, may have reduced the precision of estimates in this study, attrition is not considered seriously to limit the findings in this study.

There are other limitations to acknowledge. First, some drinking behaviours were not assessed at all survey time points, which limits the opportunity to draw conclusions about historical trends of change. Second, variation in how questions were asked in the three surveys also makes it necessary to be cautious when interpreting the results.

The validity of the CAGE as a measure of problem drinking has been debated, but our findings on problematic drinking (or alcohol misuse) in HUNT are consistent with previous findings of alcohol abuse based on other diagnostic tools (DSM-IIIR and CIDI) [52]. Skogen et al. (2011) investigated the concurrent validity and psychometric properties of the CAGE by using data from HUNT1 and HUNT2 and concluded that the internal reliability of the CAGE was adequate [53]. Findings also suggested a better concurrent validity in women than in men, which stands in contrast to Dhalla and Kopec (2007), who found that the CAGE did not perform well among white women in a primary care setting [37].

## Conclusions

Adult drinking patterns between 1984 and 2008 have changed in many respects. In the current study most changes were in the same direction for men and women, but gender differences in drinking declined. Women generally moved towards men’s drinking patterns, but changes were age- specific and varied depending on which drinking behavior and which beverage was analyzed. Future attempts to prevent or reduce the negative consequences of alcohol drinking should give greater attention to gender- and age-specific changes in drinking patterns.

## Abbreviations

H1, HUNT 1 conducted between 1984–1986; H2, HUNT 2 conducted between 1995–1997; H3, HUNT 3 conducted between 2006–2008; HUNT, The Nord-Trøndelag Health Study; Q1, Self report questionnaire no. 1; Q2, Self report questionnaire no. 2

## Additional file

Additional file 1: Supplementary: Classification of alcohol measures in HUNT1 (1984-86), HUNT2 (1995-97), HUNT3 (2006-08), questionnaires (Q1-Q2). (DOC 33 kb)
