# Supplementary material for: Gender differences and gender convergence in alcohol use over the past three decades (1984–2008), The HUNT Study, Norway
Source: BMC Public Health. 2016 Aug 5;16:723. doi: 10.1186/s12889-016-3384-3 (PMC4974746; doi:10.1186/s12889-016-3384-3)
Supplement: Additional file 1: — Supplementary: Classification of alcohol measures in HUNT1 (1984-86), HUNT2 (1995-97), HUNT3 (2006-08), questionnaires (Q1-Q2). (DOC 33 kb) [file 12889_2016_3384_MOESM1_ESM.doc]

**Supplementary: Classification of alcohol measures in HUNT1 (1984-86), HUNT2 (1995-97), HUNT3 (2006-08), questionnaires (Q1-Q2)**

| **Outcomes** | **HUNT1** | **HUNT2** | **HUNT3** |
| --- | --- | --- | --- |
| **Recent drinking** | Q2 How often did you drink alcohol (beer, wine or spirits) during the LAST 14 DAYS? 5 response alternatives  Recent= at least one drinking occasion during past 14 days | Q1 How many times a month do you normally drink alcohol? (open response) Q1  How many glasses of beer, wine or spirits do you usually drink in the course of two weeks?  Recent=those who reported any drinking occasions and simultaneously reported their 2-weeks amount of alcoholic beverages | Q1 Have you ever been drinking alcohol during the past 4 weeks?  Q1 How many glasses of beer, wine or spirits do you usually drink in the course of two weeks?  Recent= those who reported any drinking occasions and simultaneously reported their 2-weeks amount of alcoholic beverages |
| **Abstaining** | Q2 How often did you drink alcohol (beer, wine or spirits) during the LAST 14 DAYS? 5 response alternatives  Abstaining= those who reported total abstention from alcohol | Q1 Concerning alcohol: Do you totally abstain from alcohol (yes or no response)  Abstaining= yes | Q1 About how often have you been drinking alcohol during past 12 months  (8 response alternatives)  Abstaining= those who reported lifetime or last year abstention |
| **Intoxication**  (among recent drinkers) | Q2 If you drank alcohol during the past 14 days, have you on any occasion felt intoxicated? (yes or no response)  Intoxication= yes | No questions | Q1 Have you ever been drinking alcohol during the past 4 weeks? (yes or no response)  If YES, Did you drink so much that you felt very intoxicated (drunk)? |
| **Lifetime problematic drinking** | Q2 Have there been periods in your life when yo drank excessively or too much? Only one item was used (not reported in tables) | Q2 CAGE instrument , 4 items, max score=4  Problematic drinking claims at least “yes” on two or more of the 4 CAGE items | Q2 CAGE instrument , 4 items, max score=4  Problematic drinking claims at least “yes” on two or more of the 4 CAGE items |
| **Alcohol consumption** | No questions | Q1 How many glasses of beer, wine or spirits do you usually drink in the course of two weeks? (Do not include low-alcohol beer. Write 0 if less than once a month.) Consumption was defined as any consumption above zero past 14 days | Q1 How many glasses of beer, wine or spirits do you usually drink in the course of two weeks? (Do not include low-alcohol beer. Write 0 if less than once a month) Consumption was defined as any consumption above zero past 14 days |
